# Anti-leukemic activity and tolerability of anti-human CD47 monoclonal antibodies

**DOI:** 10.1038/bcj.2017.7

**Published:** 2017-02-24

**Authors:** E C Pietsch, J Dong, R Cardoso, X Zhang, D Chin, R Hawkins, T Dinh, M Zhou, B Strake, P-H Feng, M Rocca, C Dos Santos, X Shan, G Danet-Desnoyers, F Shi, E Kaiser, H J Millar, S Fenton, R Swanson, J A Nemeth, R M Attar

**Affiliations:** 1 Oncology Discovery, Janssen Research and Development, Spring House, PA, USA; 2 Biologics Research, Janssen Research and Development, San Diego, CA, USA; 3 Biologics Research, Janssen Research and Development, Spring House, PA, USA; 4 Biologics Toxicology, Janssen Research and Development, Spring House, PA, USA; 5 Division of Hematology and Oncology, Department of Medicine, Perelman School of Medicine, University of Pennsylvania, Philadelphia, PA, USA; 6 Pharmaceutical Development and Manufacturing Sciences, Janssen Research and Development, Malvern, PA, USA

## Abstract

CD47, a broadly expressed cell surface protein, inhibits cell phagocytosis via interaction with phagocyte-expressed SIRPα. A variety of hematological malignancies demonstrate elevated CD47 expression, suggesting that CD47 may mediate immune escape. We discovered three unique CD47-SIRPα blocking anti-CD47 monoclonal antibodies (mAbs) with low nano-molar affinity to human and cynomolgus monkey CD47, and no hemagglutination and platelet aggregation activity. To characterize the anti-cancer activity elicited by blocking CD47, the mAbs were cloned into effector function silent and competent Fc backbones. Effector function competent mAbs demonstrated potent activity *in vitro* and *in vivo,* while effector function silent mAbs demonstrated minimal activity, indicating that blocking CD47 only leads to a therapeutic effect in the presence of Fc effector function. A non-human primate study revealed that the effector function competent mAb IgG1 C47B222-(CHO) decreased red blood cells (RBC), hematocrit and hemoglobin by >40% at 1 mg/kg, whereas the effector function silent mAb IgG2σ C47B222-(CHO) had minimal impact on RBC indices at 1 and 10 mg/kg. Taken together, our findings suggest that targeting CD47 is an attractive therapeutic anti-cancer approach. However, the anti-cancer activity observed with anti-CD47 mAbs is Fc effector dependent as are the side effects observed on RBC indices.

## Introduction

CD47, also known as integrin-associated protein, is a ubiquitously expressed 50 kDa cell surface transmembrane Ig superfamily member. CD47 interacts with integrins (for example, αvβ3, αIIbβ3, and α2β1), thrombospondin-1, and serves as a ligand for signal regulatory protein alpha (SIRPα).^1^ Due to its multiple interaction partners, CD47 mediates a variety of biological processes, including leukocyte adhesion and migration, T-cell activation, apoptosis and phagocytosis.^2, 3^

Phagocytosis is a complex multi-step process that facilitates the removal of apoptotic as well as IgG- or complement-opsonized cells, and is enabled and balanced by positive and negative regulatory receptor-ligand interactions between target and effector cells.^4^ Studies with erythrocytes, platelets and leukocytes identified the CD47-SIRPα interaction as a negative regulator of phagocytosis.^5, 6, 7, 8^

Increased expression of CD47 has been shown in a variety of solid (ovarian, bladder, breast, glioma and glioblastoma) and hematological malignancies (acute myeloid leukemia, lymphoblastic leukemia, and Non-Hodgkin lymphoma) and elevated expression negatively correlates with clinical outcome.^9, 10, 11, 12^ Furthermore, CD47 has been identified as a cancer stem cell marker in both leukemias and solid tumors.^13, 14, 15, 16^ Therefore, therapeutic targeting of CD47 may have widespread application in numerous cancers, as overexpression of CD47 may allow cancer cells to co-opt CD47-SIRPα signaling and evade phagocytosis-mediated elimination.^17^ In support, several preclinical cancer models using established cancer cell lines and primary cancer cells demonstrated that anti-human CD47 mAbs as well as human SIRPα-Fc proteins mediated phagocytosis of cancer cells by human and mouse macrophages *in vitro*, and mediated *in vivo* anti-tumor efficacy.^11, 12, 18, 19^

Although targeting CD47 represents a unique mechanism of action and may have broad applicability across various cancers, the ubiquitous nature of CD47 presents a therapeutic challenge. The impact of a monoclonal antibody with an effector function competent Fc region that could mediate antibody-dependent cellular cytotoxicity (ADCC), complement-dependent cytotoxicity (CDC) and antibody-dependent cellular phagocytosis (ADCP) on individual cells and tissues is not fully understood. Moreover, anti-CD47 antibodies have been reported to cause platelet aggregation and red blood cell hemagglutination.^20, 21, 22^

Herein, we describe the generation and characterization of anti-CD47 monoclonal antibodies that specifically bind to CD47, block CD47-SIRPα, and do not induce hemagglutination and platelet aggregation activity. Initially, the anti-CD47 mAbs were tested using *in vitro* and *in vivo* AML models. AML is difficult to treat due to a combination of biological heterogeneity and patient-related risk factors such as age or co-morbidities, resulting in poor long-term overall survival.^23^ Targeting of surface markers, such as CD47, promises a novel therapeutic approach in AML. While our studies provide evidence of the anti-leukemic effects of targeting CD47 with a monoclonal antibody, they also demonstrate that the efficacy and tolerability of anti-CD47 mAbs are dependent on Fc effector function.

## Materials and methods

### Patient samples and cell lines

Peripheral blood/bone marrow samples were obtained from AML patients (Supplementary Table 1) after informed consent in accordance with a protocol approved by the Institutional Review Board at the University of Pennsylvania. Jurkat, HL60, Kasumi-3, MV4-11 and Wil2-S cells were purchased from ATCC (Manassas, VA, USA). Jurkat, MV4-11 and Wil2-S cells were cultured in RPMI1640 (Invitrogen, Carlsbad, CA, USA) supplemented with 10% FBS (Invitrogen). Kasumi-3 cells were cultured in RPMI1640 supplemented with 20% FBS and HL60 were cultured in IMDM supplemented with 20% FBS. All cells were maintained at 37 °C in a humidified atmosphere containing 5% CO2.

### Generation of anti-CD47 antibodies

Anti-CD47 monoclonal antibodies were identified using hybridoma and phage display technologies. They were further engineered for human framework adaptation or affinity improvement. Detailed descriptions on antibody generation, and their characterization in terms of binding affinity, epitope/paratope description in atomic details, SIRPα blocking activity, hemagglutination, and platelet aggregation can be found in the Supplementary section.

### Phagocytosis

CD14^+^ monocytes were isolated from leukopak purified healthy donor peripheral blood mononuclear cells (PBMCs; Biological Specialty Corporation, Colmar, PA, USA) by negative depletion using a CD14 isolation kit without CD16 depletion (Stem Cell Technologies, Cambridge, MA, USA). Monocytes were plated at 0.1 × 10^6^ cells/cm^2^ in X-VIVO 10 growth medium (Lonza, Basel, Switzerland) supplemented with 10% FBS and 25 ng/ml M-CSF (R&D Systems, Minneapolis, MN, USA) and were cultured for 7 days for differentiation to macrophages. 50 ng/ml IFN-γ (R&D Systems) was added during the final 24 h of differentiation. Subsequently, macrophages (1 × 10^5^) were plated at a 1:1 ratio with GFP+ T-ALL (Jurkat) or CFSE+ labeled AML (Kasumi-3, MV4-11, or HL60) cells in the presence of anti-CD47 mAbs. Cells were then incubated at 37 °C for 90 min and macrophages were stained with an anti-human CD11b (APC conjugated; eBiosciences, San Diego, CA, USA) for 30 min followed by two washing steps with stain buffer (BD Biosciences, San Jose, CA, USA). Cells were analyzed by flow cytometry (MacsQuant, Miltenyi Biotec, San Diego, CA, USA) to determine tumor cells alone (CFSE+/GFP+, CD11b−), macrophages alone (CFSE−/GFP−, CD11b+), and phagocytosed tumor cells (CFSE+/GFP+, CD11b+). Percent phagocytosis was determined by the following equation: (phagocytosed tumor cells)/(phagocytosed tumor cells + tumor cells alone) × 100%.

### Antibody-dependent cellular cytotoxicity

Healthy donor PBMCs (Biological Specialty Corporation) were plated in X-VIVO 10 growth media (Lonza) supplemented with 10% FBS 16 h prior to initiating ADCC assays. The day of the assay, 10 000 calcein AM labeled HL60 target cells were incubated with healthy donor PBMCs (2 × 10^5^) in the presence of varying concentrations of anti-CD47 antibodies for 3 h at 37 °C. Calcein AM release was measured by fluorescence at 485–535 nm with a SpectraMax M5 multimode plate reader (Molecular Devices, LLC, Sunnyvale, CA, USA). Specific lysis was determined by normalizing the data to maximal (Triton X-100 mediated) and minimal (effector cells alone) lysis.

### Complement-dependent cytotoxicity (CDC)

A total of 50 000 Wil2-S cells were plated in growth media in the presence of anti-CD47 mAbs and incubated for 30 min at 37 °C. Subsequently, complement preserved human serum was added and cells were incubated at 37 °C for 2 h. CellTiter-Glo Reagent (Promega, Madison, WI, USA) was added, and luminescence was recorded on the 2104 EnVision Multilabel reader (Perkin Elmer, Waltham, MA, USA). To assess maximal lysis 2% Triton X-100 was added to control cells. Specific lysis was calculated by the following equation: (specific lysis = ((experimental release−spontaneous release)/(maximum release−spontaneous release)) × 100).

### Mouse models of human AML

*In vivo* studies with human AML cell lines were conducted with non-irradiated NOD.Cg-Prkdc^scid^ Il2rg^tm1Wjl^/SzJ (NSG) mice. Female mice (4–6 week old) were purchased from the Jackson Laboratory (Bar Harbor, ME, USA). All animal procedures were approved by the Janssen Research and Development Institutional Animal Care and Use Committee (IACUC). HL60, Kasumi-3, and MV4-11 AML cell lines (Supplementary Table 2) were injected intravenously (IV), and antibody treatment was administered by intraperitoneal (IP) injection. Engraftment of leukemic cells was measured by relative percentage of human CD45+ cells in the bone marrow, spleen and/or peripheral blood. Primary AML patient sample models were conducted in irradiated NSG mice. NSG mice were produced at the University of Pennsylvania using breeders obtained from Jackson Laboratory. 24 h prior to AML injection (IV), mice were sublethally irradiated (275 cGy delivered at 100 cGy/min using a Gammacell 40 Exactor, MDS Nordion, Kanata, ON, Canada). All animal procedures were conducted in accordance to a protocol approved by the IACUC at the University of Pennsylvania. Engraftment of leukemic cells was measured by relative percentage of human CD45+CD3- cells in the bone marrow and spleen.

### Non-human primate (NHP) study

A NHP study was conducted at Charles River Laboratories (Reno, NV, USA) in accordance with standard operating procedures. All animal procedures were approved by the Institutional Animal Care and Use Committee. Experimentally non-naive, socially housed, female cynomolgus monkeys of Chinese origin (*n*=4/group, 2–5 years of age and weighing 2.8 to 3.7 kg at the onset of the study) were administered anti-CD47 mAbs on days 1 and 8 by a slow bolus IV injection. Animals were observed at least twice daily. Peripheral blood was collected for hematology assessment, RBC receptor occupancy and clinical pathology evaluations as indicated in the manuscript. Monkeys were returned to the animal colony at the completion of the study.

## Results

### Discovery and characterization of anti-CD47 antibodies

A panel of unique anti-CD47 antibodies were discovered using hybridoma and phage display technologies and were engineered as human IgG2σ, which has an effector function silent Fc.^24^ The mAbs were evaluated in terms of SIRPα-blocking activity, binding affinity to human and cynomolgus monkey (cyno) CD47, epitope binning groups, and hemagglutination activity. The commercially available anti-CD47 mAb B6H12.2 (CD47-SIRPα blocking; ATCC) was engineered with the human IgG2σ Fc and used for comparison in the above assays. The characteristics of 23 mAbs (20 from hybridoma and 3 from phage display methods) and B6H12.2 are summarized in Supplementary Table 3. The majority of these mAbs were potent SIRPα blockers with EC50 values below 1 μg/ml and demonstrated cross-reactivity to cyno CD47 with binding affinity within 5-fold of their affinity to human CD47. The mAbs were classified into four epitope groups based on competition binding studies. Fifteen of the 23 mAbs were found to induce hemagglutination (for example, IgG2σ C47B98; Supplementary Figures 1a and b), which did not appear to be associated with any of the other properties tested.

On the basis of the desired characteristics, such as potent SIRPα-blocking activity, cross-reactivity to cyno CD47, and lack of induction of hemagglutination, we identified two mAbs, namely C47B116 (hybridoma derived) and C47B91 (phage derived), for further optimization via human framework adaptation (HFA) and affinity maturation (AM), respectively. The C47B116 HFA and C47B91 AM variants were expressed as effector function silent IgG2σ and evaluated again for the above properties as well as platelet aggregation. C47B157 and C47B161, derived from C47B116, and C47B222, derived from C47B91, were selected as lead candidates for further evaluation. These mAbs showed potent SIRPα-blocking activity, good binding affinity to both human and cyno CD47 (1–4 nM), and did not induce hemagglutination or platelet aggregation (Table 1, Supplementary Figures 1c and 2a–d).

**Figure 1:**
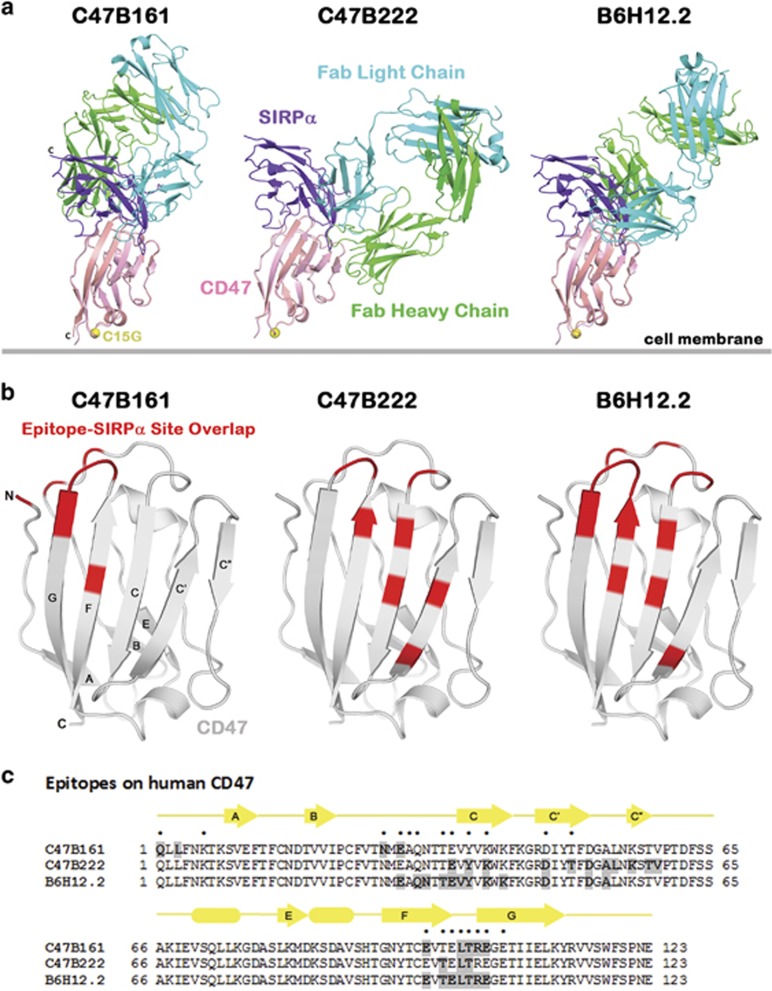
C47B161, C47B222 and B6H12.2 Fabs have steric clashes with SIRPα. (**a**) Structural overlay of Fab/CD47 complexes onto the SIRPα/CD47 complex showing regions of clash between Fab and SIRPα. The overlay was achieved by superposition of equivalent CD47 Cα atoms in both complexes. The membrane-proximal C15G mutation that was introduced to prevent CD47 aggregation is shown as a yellow sphere. (**b**) Regions of overlap between each epitope and the SIRPα binding site (red areas). The structure of CD47 from the C47B222 complex was used in (**b)**. (**c**) Epitope regions of C47B161, C47B222 and B6H12.2. The epitope residues are shaded and SIRPα binding residues are marked with a dot above the human CD47 ECD sequence. The secondary structure of CD47 is shown above the sequence with arrows and cylinders representing β-strands and helices, respectively.

The detailed atomic interactions of C47B161, C47B222 and B6H12.2 with the human CD47 extracellular domain were determined using crystal structures of Fab/CD47 complexes (Supplementary Table 4, and Supplementary Figures 3 and 4). The C47B157 complex structure was not determined because C47B157 and C47B161 have identical CDRs and are expected to bind to the same epitope. The overlays of the Fab/CD47 structures onto the SIRPα/CD47 structure (PDB code 2JJS;^25^) demonstrate that heavy and light chains of all three antibodies have steric clashes with SIRPα and block its interaction with CD47 (Figure 1a). The epitope regions that overlap with the SIRPα binding site are located in the β-sheet and loop regions of CD47. The B6H12.2 epitope has a larger overlapping area with the SIRPα site than the other two epitopes (Figure 1b, regions of overlap marked in red). Specifically, the CD47 regions of overlap for each antibody are (Figure 1c): C47B161: residues Q1, N27, E29, E97, and L101-E104; C47B222: residues E35, Y37, K39, D46, T49, T99, L101 and T102; B6H12.2: residues E29, Q31, T34, E35, Y37, K39, D46, E97 and T99-E104.

### Effector function competent (IgG1) anti-CD47 mAbs mediate ADCP and ADCC, while effector function silent mAbs (IgG2σ) have limited *in vitro* activity

To characterize the role of Fc function on the activity of anti-CD47 antibodies, three lead mAbs, IgG2σ C47B157, C47B161 and C47B222, were converted to an effector function competent human IgG1 Fc format and were evaluated *in vitro* and subsequently *in vivo*. As a reference antibody, the anti-CD47 B6H12.2 was generated with either a human IgG2σ or IgG1.

**Figure 2:**
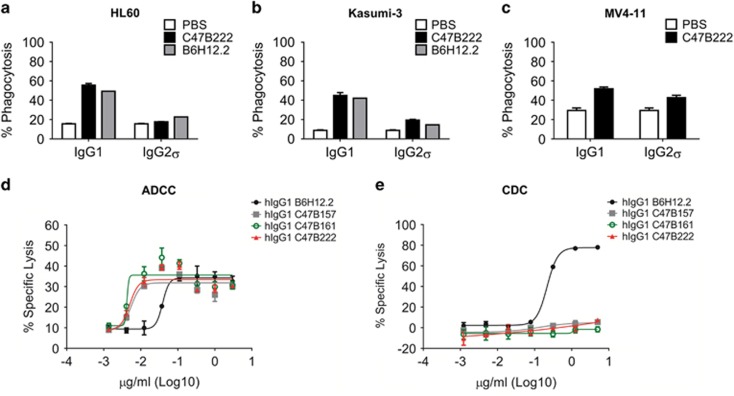
IgG1 anti-CD47 mAbs mediate ADCP and ADCC activity. ADCP of HL60 (**a**), Kasumi-3 (**b**), or MV4-11 (**c**) cells by human PBMC-derived macrophages in response to IgG1/IgG2σ anti-CD47 mAbs. (**d**) ADCC of human PBMC against HL60 target cells in response to increasing concentrations of IgG1 anti-CD47 mAbs. (**e**) CDC against WIL2-S target cells in the presence of human complement in response to increasing concentrations of IgG1 anti-CD47 mAbs.

Initially, *in vitro* phagocytosis assays were conducted with CD47 expressing T-ALL Jurkat target cells stably transfected with GFP (Supplementary Figure 5a) and human PBMC-derived macrophages. IgG1 C47B157, C47B161 and C47B222 mAbs induced phagocytosis in a concentration-dependent manner comparable to IgG1 B6H12.2 (Supplementary Figure 5b). Maximal phagocytosis was achieved at 5 μg/ml (Supplementary Figure 5b), and higher antibody concentrations did not further impact observed phagocytosis (data not shown). In contrast, effector function silent IgG2σ C47B157, C47B161, C47B222 and B6H12.2 had a minimal effect on phagocytosis (Supplementary Figure 5b). In subsequent experiments, we characterized the ADCP activity of representative anti-CD47 mAbs, IgG1 and IgG2σ C47B222, with CFSE labeled CD47 expressing HL60, Kasumi-3, and MV4-11 AML cell lines (Supplementary Figure 5c). C47B222 and B6H12.2 consistently induced significant phagocytosis of these cells as IgG1 (1.8-5 fold over PBS control), but demonstrated markedly reduced activity as IgG2σ (1.2-2.2 fold; Figures 2a–c). These results demonstrated that blocking CD47 only lead to significant phagocytosis in the presence of a phagocytic stimulus, that is, Fc effector function.

ADCC and CDC assays were conducted with the effector function competent IgG1 C47B157, C47B161 and C47B222 mAbs to further characterize the anti-leukemic activity of these antibodies. In ADCC assays that utilized PBMCs and HL60 target cells, similar average maximal lysis of HL60 cells was observed in response to IgG1 C47B157 (40.7%), C47B161 (42%), C47B222 (37.3%) and B6H12.2 (38.4%), with half maximal effective concentration values (EC50) of 0.0085 μg/ml, 0.0067 μg/ml, 0.0068 μg/ml and 0.0478 μg/ml, respectively (Figure 2d). CDC activity was observed for effector function competent IgG1 B6H12.2 with an average EC50 of 0.2 μg/ml, whereas no CDC activity was observed for IgG1 C47B157, C47B161, and C47B222 (Figure 2e). Differences in CDC activity between IgG1 B6H12.2 and C47B157, C47B161 and C47B222 may be due to differences in mAb off-rate and the epitope recognized by the mAbs.^26^ In summary, the identified effector function competent anti-CD47 mAbs showed potent ADCP and ADCC activity, but no CDC activity.

### Effector function competent (IgG1) and silent (IgG2σ) anti-CD47 mAbs demonstrate different anti-leukemic activity *in vivo*

We next characterized the *in vivo* efficacy of IgG1 and IgG2σ anti-CD47 mAbs using three xenograft models of human AML. HL60, MV4-11 and Kasumi-3 cells were transplanted intravenously into NSG mice. Six days after transplantation, treatment with the anti-CD47 mAbs was initiated and leukemic cell growth was assessed as the percentage of human CD45+ cells present in the peripheral blood.

Throughout the study, no changes in body weights of treated mice were observed (data not shown).

**Figure 3:**
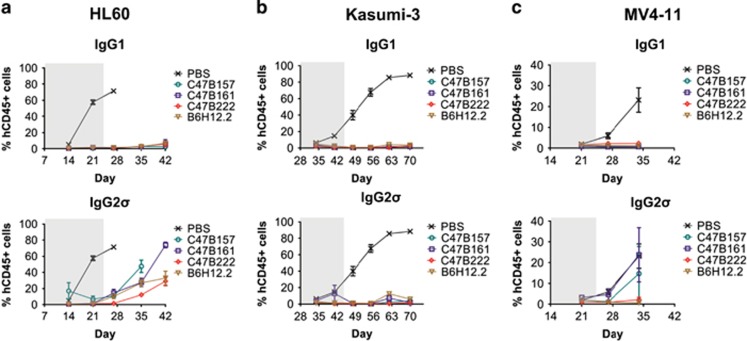
*In vivo* activity of IgG1 and IgG2σ anti-CD47 mAbs in AML xenograft models. (**a**) Ten million HL60 cells were implanted into NSG mice (*n*=5/group) on day 0 and mice received twice weekly treatments for 3 weeks (10 mg/kg) starting day 6 after implant (final day 23; grey-shaded area). Control mice were killed on day 27. (**b**) Ten million Kasumi-3 cells were implanted into NSG mice (*n*=5/group) on day 0 and mice received twice weekly treatments (10 mg/kg) for 6 weeks, starting day 6 after implant (final day 44; grey-shaded area). (**c**) Five million MV4-11 cells were implanted into NSG mice (*n*=5/group) on day 0 and mice received twice weekly treatments (10 mg/kg) for 3 weeks, starting day 6 after implant (final day 23; grey-shaded area). Control mice were killed on day 34. (**a**–**c**) The graphs show peripheral blood leukemic burden (human CD45+ cells) over time, which was monitored weekly, starting on day 14 (HL60 and Kasumi-3) or day 21 (MV4-11).

As shown in Figure 3, treatment with 10 mg/kg IgG1 C47B157, C47B161 and C47B222 potently suppressed leukemia outgrowth (0.74–2.78% CD45+ cells) across all models vs PBS control treated mice (23.2–88.5% CD45+ cells). In contrast, the effector function silent IgG2σ C47B157, C47B161, C47B222 and B6H12.2 mAbs displayed variable anti-leukemic activity. While all IgG2σ anti-CD47 mAbs suppressed leukemia growth similar to IgG1 anti-CD47 mAbs in the Kasumi-3 model (Figure 3b), anti-leukemic effects of the IgG2σ anti-CD47 mAbs were less pronounced in the HL60 and MV4-11 models (Figures 3a and c). In the HL60 model, IgG2σ C47B157, C47B161 and C47B222 initially prevented leukemic outgrowth (10.84%, 10.48% and 1.62% CD45+ cells, respectively) compared to the PBS control (71.42%) at the time of control sacrifice. However, between Day 27 and 42, leukemic cells expanded in the peripheral blood and approached levels (40–80% CD45+ cells) similar to those observed in the PBS control. In the MV4-11 model (Figure 3c), IgG2σ C47B222 and B6H12.2 limited leukemic burden (2.18% and 0.7% CD45+ cells, respectively) compared to PBS control (23.15% CD45+ cells), while IgG2σ C47B157 and C47B161 had a minimal impact (14.75% and 23.76% CD45+ cells, respectively).

These initial *in vivo* studies demonstrated that the effector function competent IgG1 anti-CD47 mAbs consistently mediated potent anti-leukemic activity. With respect to the IgG2σ-based mAbs, C47B222 demonstrated the most consistent anti-leukemic activity *in vivo*.

### Targeting CD47 with an effector function silent anti-CD47 mAb does not prolong survival

Due to its anti-leukemic activity in initial studies, subsequent studies focused on C47B222-based IgG1 and IgG2σ antibodies. IgG1 and IgG2σ C47B222 produced from stable CHO cell lines were used and referred to as IgG1 C47B222-(CHO) and IgG2σ C47B222-(CHO), respectively.

**Figure 4:**
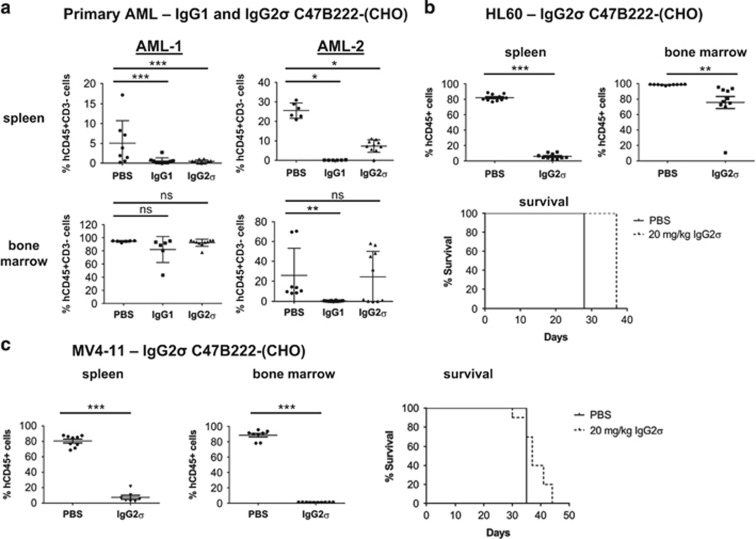
*In vivo* activity of IgG1 and IgG2σ C47B222-(CHO) in primary AML and xenograft mouse models. (**a**) NSG mice were implanted with the indicated AML patient samples. Bone marrow aspirates were analyzed 5 weeks later for engraftment, mice were randomized and treatment was initiated (*n*=10/group). IgG1 and IgG2σ C47B222-(CHO) were administered twice weekly (10 mg/kg) for 3 weeks. BM and spleen were collected on day 21 and analyzed via FACS to assess leukemic cell burden (hCD45+CD3− cells). (**b**) Ten million HL60 cells were implanted into NSG mice (*n*=10/group) on day 0 and mice received twice weekly treatments (20 mg/kg), starting day 6 after implant until study end. (**c**) Five million MV4-11 cells were implanted into NSG mice (*n*=10/group) on day 0 and mice received twice weekly treatments (20 mg/kg), starting day 6 after implant until study end. For both (**b**) and (**c**), survival was monitored, and bone marrow and spleen were collected on day 28 (HL60) and day 35 (MV4-11) and analyzed via FACS to assess leukemia burden (hCD45+ cells). Increased life span (ILS) was calculated as follows: ((treatment median survival−control median survival)/control median survival) × 100; Statistical significance was assessed by unpaired *t*-test vs PBS control (*P<0.05, **P<0.01 and ***P<0.0001; NS, non-significant).

*In vivo* studies using primary AML patient sample or cell line-engrafted NSG mice were conducted to assess the effects of IgG1 and IgG2σ C47B222-(CHO) on leukemic cell engraftment in bone marrow and spleen. NSG mice were engrafted with two primary AML patient samples (AML-1 and AML-2). IgG2σ C47B222-(CHO) significantly reduced leukemic cell growth in spleen compared to PBS control-treated mice (Figure 4a) in both models when administered at 10 mg/kg twice weekly until study end, but did not reduce bone marrow tumor burden. Similarly, IgG1 C47B222-(CHO) effectively cleared splenic leukemia burden in both models. However, IgG1 C47B222-(CHO) demonstrated marked differences in clearing leukemic cells in the bone marrow of mice engrafted with primary samples AML-1 and AML-2. Specifically, IgG1 C47B222-(CHO) lead to near complete elimination of leukemic cells in the bone marrow of AML-2 engrafted mice, but not AML-1 engrafted mice (Figure 4a). The observed differential activity of IgG1 C47B222-(CHO) in clearing bone marrow leukemic burden may be dependent on the amount of disease present at the time of initiating treatment. Bone marrow aspirates conducted at the time of randomization into treatment groups demonstrated that the mean percentage of CD45+ cells in AML-1 and AML-2-engrafted mice was 44% (range 12.5–82.5%) and 24% (range 0.8–75.4%), respectively.

To further characterize the contribution of effector function to anti-leukemic activity, subsequent studies focused on characterizing IgG2σ C47B222-(CHO) in AML xenograft models. The antibody was administered at 20 mg/kg twice weekly until study end and no changes in body weight of the mice were observed (data not shown). Similar to the primary AML models, IgG2σ C47B222-(CHO) significantly cleared splenic leukemia burden in NSG mice engrafted with HL60 cells and demonstrated a significant albeit small decrease in bone marrow leukemia burden (98.6 vs 75.6% CD45+ cells PBS vs IgG2 C47B222-(CHO); Figure 4b).

Consequently, the observed increase in life span (ILS) in response to IgG2σ C47B222-(CHO) treatment was not significant (ILS = 32% *P*=1). Similarly, treatment of mice bearing MV4-11 did not yield a significant survival advantage in response to IgG2σ C47B222-(CHO) (Figure 4c). Although IgG2σ C47B222-(CHO) significantly cleared leukemia burden in spleen and bone marrow the observed ILS was 6% (*P*=1), likely due to leukemic cell growth in other organs as suggested by the observations of facial tumors, hind limb paralysis and distended abdomens.

Collectively, blocking CD47 in the absence of effector function, did not result in efficient clearance of AML disease and did not provide a survival advantage, while effector function competent IgG1 C47B222-(CHO) demonstrated anti-leukemic efficacy. However, efficacy may be dependent on tumor burden and on the microenvironment (bone marrow vs spleen) at the time of initiating treatment.

### IgG1 anti-CD47 mAb impacts red blood cell indices in a non-human primate study

**Figure 5:**
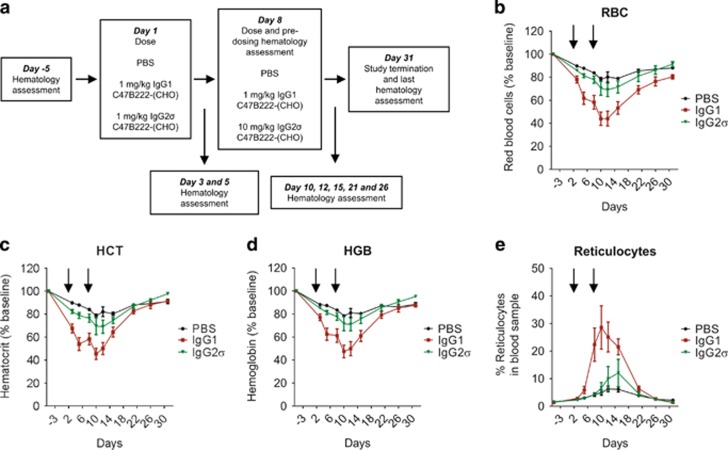
Non-human primate study with IgG1 and IgG2σ C47B222-(CHO). (**a**) Non-naive female cynomolgus monkeys were treated with anti-CD47 mAbs as detailed in schematic (*n*=4/group). Graphs summarize changes in red blood cell (**b**), hematocrit (**c**), hemoglobin (**d**), and reticulocyte levels (**e**) in response to treatment with IgG1 and IgG2σ C47B222-(CHO). Arrows above graphs indicate dosing with mAbs.

Because CD47 is a ubiquitously expressed protein, effects on normal tissues of anti-CD47 antibodies could be dose limiting. A non-human primate study was conducted to further explore the impact of targeting CD47. Cynomolgus monkeys were treated by intravenous injection with PBS (control group), the effector function competent IgG1 C47B222-(CHO) or effector function silent IgG2σ C47B222-(CHO) (Figure 5a). IgG1 C47B222-(CHO) was administered at 1 mg/kg on study days 1 and 8, while IgG2σ C47B222-(CHO) was dosed at 1 mg/kg on study day 1 and 10 mg/kg on study day 8. A substantial drop in red blood cell number (~40% compared to concurrent controls; Supplementary Table 5), hematocrit and hemoglobin was observed in response to 1 mg/kg IgG1 C47B222-(CHO). A second dose of IgG1 C47B222-(CHO) lead to an additional, albeit small, drop in red blood cell indices (Figures 5b–d). Notably, a compensatory response was mounted in response to the red blood cell drop as evidenced by the increases in reticulocytes (Figure 5e). Red blood cell indices started to recover on study day 15, and returned to levels observed in the PBS control by study day 31. Treatment with 1 and 10 mg/kg IgG2σ C47B222-(CHO) minimally impacted red blood cell count (~13% reduction compared to concurrent controls; Supplementary Table 5), hematocrit and hemoglobin values. Treatment did not affect other hematology parameters, clinical chemistry or coagulation. Receptor occupancy analysis demonstrated that the majority of RBCs were bound to IgG1 and IgG2σ C47B222-(CHO) within 1 h of treatment (data not shown).

## Discussion

CD47 overexpression has been observed in heme malignancies and solid tumors. As CD47 is a negative regulator of phagocytosis, the hypothesis that blocking CD47 could promote tumor cell elimination emerged. In support, compelling data on the anti-tumor activity of anti-CD47 mAbs (that is, B6H12.2 and Hu5F9-G4) or SIRPα-Fc construct have been reported.^9, 11, 12, 19, 27^ However, the Fc region of the anti-CD47 mAbs and SIRPα-Fc utilized in these studies consisted of murine IgG1 (B6H12.2) and human IgG4 (Hu5F9-G4 and SIRPα-Fc), both of which bind human and murine Fc receptors and mediate effector functions.^28, 29, 30, 31^ Hence, these studies did not define whether the therapeutic effect observed was due to solely blocking CD47 or to an opsonizing effect combined with CD47 blocking activity.

Herein, we describe the generation and characterization of anti-CD47 mAbs that block binding of CD47 to SIRPα and do not demonstrate hemagglutination or platelet aggregation activity. The anti-leukemic activity of these mAbs was tested *in vitro* and *in vivo* to determine the role of effector function competent and silent Fc backbones. These studies revealed that anti-leukemic activity of anti-CD47 mAbs is dependent on Fc effector function. These results are in alignment with the data generated by independent research groups. For example, an engineered high-affinity human SIRPα variant known as CV1 was shown to mediate potent *in vitro* and *in vivo* anti-tumor activity as IgG4 Fc conjugate, but not as SIRPα monomer.^32^ Similarly, a SIRPα-Fc molecule known as TTI-621 demonstrated potent anti-leukemic activity as IgG1 Fc conjugate, but not with a Fc mutated to lack effector function.^33^ Furthermore, mice that lack the SIRPα cytoplasmic tail, and hence SIRPα's inhibitory signaling ability, demonstrated equivalent growth and metastasis of B16F10 syngeneic melanoma tumor cells upon implant as wild-type mice, suggesting that disruption of CD47-SIRPα alone does not yield an anti-tumor effect.^34^ Importantly, while these studies provide evidence that altering CD47- SIRPα does not yield anti-tumor activity, these and other studies showed that blocking CD47-SIRPα inhibitory signaling lowers the threshold for phagocytosis and allowed for more efficient tumor cell elimination *in vitro* and *in vivo* in response to targeted monoclonal antibody therapies, such as trastuzumab and rituximab.^9, 32, 34^ Whether the herein described antibodies perform similar functions was not formally tested and remains to be determined.

The effector function competent anti-CD47 mAbs described herein demonstrated robust ADCP and ADCC *in vitro* and mediated anti-leukemic activity *in vivo*. Subsequent to treatment with these antibodies, minimal levels of leukemic cells were detected in the peripheral blood of mice engrafted with HL60, MV4-11 and Kasumi-3 AML cells. Likewise, treatment of mice engrafted with a primary AML sample (AML-2) with effector function competent IgG1 C47B222-(CHO) resulted in clearance of leukemic cells in spleen and bone marrow. However, studies with a different primary AML patient sample (AML-1), revealed that IgG1 C47B222-(CHO) affected only splenic, but not bone marrow leukemia burden. Since the anti-leukemic activity of IgG1 C47B222-(CHO) anti-CD47 mAb does not seem to correlate with CD47 receptor density across the different AML models (data not shown), the differential activity of IgG1 C47B222-(CHO) may potentially be ascribed to bone marrow engraftment at the time of treatment start. For example, bone marrow engraftment for the AML-1 and AML-2 primary sample models was on average 44 and 24%, respectively. Infiltration of the disease into the bone marrow may have reprogramed the microenvironment to not allow for clearance of antibody targeted leukemic cells, as suggested by a recent publication on the anti-leukemic activity of alemtuzumab in a humanized model of treatment refractory B cell leukemia.^35^ In this model, alemtuzumab efficiently reduced leukemia burden in a macrophage-dependent manner in peripheral blood, spleen and liver, but not the bone marrow. Resistance to clearance of bone marrow leukemic cells was regulated by leukemia cell-secreted PGE2 and leukemia cell-expressed inhibitory FcγRIIB. Combination of antibody therapy with chemotherapy, specifically cyclophosphamide, was shown to overcome resistance by inducing a leukemic cell cytokine secretory response that promoted macrophage infiltration and activation.^35^ This study suggests that the anti-leukemic activity of anti-CD47 mAbs may benefit from combination with chemotherapeutic agents that enhance the activity of macrophages in the tumor microenvironment.

Although effector function competent anti-CD47 mAbs, such as IgG1 C47B222-(CHO), demonstrate promising efficacy, a non-human primate study conducted with IgG1 and IgG2σ C47B222-(CHO) revealed that tolerability of anti-CD47 mAbs may be limited by effector function. Specifically, treatment of cynomolgus monkeys with two doses of 1 mg/kg IgG1 C47B222-(CHO) caused >40% reduction in RBC indices. In contrast, administration of IgG2σ C47B222-(CHO) at 1 and 10 mg/kg had minor impacts (~13% reduction) on RBC parameters. Cynomolgus monkeys mounted a compensatory response as evidenced by an increase in reticulocytes in response to IgG1 C47B222-(CHO) treatment that ultimately resulted in a return of red blood cell indices to levels comparable to PBS control upon ceasing treatment. However, the effects of continued treatment as well as higher-dose levels are not known. In addition, it is not clear if an optimal dosing strategy could be achieved that provides a therapeutic window with limited toxicity. Recent evidence by Liu *et al.* and Piccione *et al.*^27, 36^ suggest that alternative treatment regiments or antibody targeting formats may circumvent the challenges associated with targeting CD47. For example, non-human primate studies conducted with an anti-CD47 mAb known as Hu5F9-G4 demonstrated that anemia was minimized and therapeutic dosing levels were achieved by administering a low priming dose to stimulate production of reticulocytes followed by a higher maintenance dose that resulted in sustained serum antibody levels.^27^ Piccione *et al.*^36^ proposed that an alternative approach to targeting CD47 could be to generate a bispecific antibody that combines a monovalent CD47 targeting arm with a tumor-specific antigen arm thereby potentially improving tumor cell selectivity and tolerability. A CD47 × CD20 antibody, which demonstrated tumor-selective binding, served as proof of concept for this approach. Interestingly, a combination effect was achieved through targeted antibody therapy and lowering the threshold for phagocytosis via CD47-SIRPα blockade.^32, 36^

In conclusion, targeting CD47 in a monospecific antibody format is challenging as anti-tumor activity and tolerability of anti-CD47 mAbs are dependent on Fc effector function. Despite these findings, CD47 remains an intriguing therapeutic target and warrants further exploration of alternative approaches to use CD47 blockade to overcome immune evasion mechanisms established by cancer cells.

## Figures and Tables

**Table 1:** Anti-CD47 mAb characteristics

*Ab ID*	*Human CD47 K*_*D*_	*Cyno CD47 K*_*D*_	*K*_*D*_ *ratio*	*SIRPα-blocking*
	*(nM)*	*(nM)*	*Cyno/Human*	*EC*_50_ *(μg/ml)*
C47B157	3.53	3.71	1.1	0.13
C47B161	2.87	2.96	1.0	0.06
C47B222	1.12	0.84	0.8	0.04
