# Supplementary material for: Anti-leukemic activity and tolerability of anti-human CD47 monoclonal antibodies
Source: Blood Cancer J. 2017 Feb 24;7(2):e536–. doi: 10.1038/bcj.2017.7 (PMC5386341; doi:10.1038/bcj.2017.7)
Supplement: Supplementary Information [file bcj20177x1.doc]

# Supplementary Information

# MATERIALS AND METHODS

**Expression and purification of soluble CD47:** The soluble extracellular domains (ECDs) of human and cynomolgus CD47 (residues 19-141) were expressed from transiently transfected HEK293 cells as secreted proteins with a C-terminal 6xHis tag. The proteins were purified via immobilized-metal affinity chromatography (IMAC) using a 5ml HisTrap column (GE Healthcare, Little Chalfont, UK) followed by a final polishing size exclusion chromatography (SEC) step on a 26/60 Superdex 200 column (GE Healthcare).

**Generation of CD47 antibodies using hybridoma technology and human frame work adaptation:** Balb/c mice were immunized with recombinant human CD47-Fc Chimera (R&D systems, Minneapolis, MN, USA) to initiate anti-CD47 antibody development using standard immunization protocols. Hybridoma clones were developed from fusions of spleen B cells from immunized mice and SP20-Bcl2 myeloma cells, and the supernatants were assayed by ELISA for antibody binding to CD47 but not to the Fc-Tag. The hybridoma supernatants showing specific binding to CD47 were further screened for binding to CD47-expressing Jurkat cells and blocking SIRPα-binding to Jurkat cells by meso scale discovery (MSD)-based assays. Hybridoma hits showing neutralization of SIRPα-binding were selected for molecular cloning of antibody V-regions. Then, V regions were amplified through PCR with pre-mixed forwards primers and reverse primers prior to infusion cloning onto murine IgG1/K constant regions. After recombinant expression in HEK293 cells, the transient transfection supernatants containing mIgG1 mAbs were re-screened in the Jurkat cell-binding and SIRPα-blocking assays. Twenty unique mIgG1 mAbs with confirmed SIRPα-blocking activity (>40% inhibition) were selected for conversion into chimeric human IgG2σ/human kappa mAbs.

For human framework adaption (HFA) of C47B116, four human heavy chain variable regions (VH) and three human light chain variable regions (VL) were designed to replace the mouse framework in in the variable region with human frameworks while keeping the CDRs intact, based on sequence homology analysis. Constructs for each VH and VL chains were generated by gene assembly and cloned into mammalian expression vectors carrying human IgG2σ. The antibodies were expressed in HEK 293Expi expression system and were purified from culture supernatants by Protein-A chromatography.

**Generation of CD47 antibodies and affinity maturation using phage display technology:** The CD47 ECD proteins were biotinylated using a 10-fold molar excess of sulfo-NHS-LC-Biotin (Pierce, Rockford, IL, USA) for phage panning experiments. In-house de novo phage libraries have been described in detail[^1^](#_ENREF_1). Three de novo phage libraries displaying Fab variants on phage coat protein pIX were panned against biotinylated human CD47 ECD in the dimeric form for three rounds according to standard protocols. Fab proteins were produced and captured onto ELISA plates by a polyclonal anti-Fd (CH1) antibody. Biotinylated CD47 ECD was added at the desired nM concentration, and the bound biotinylated CD47 was detected by HRP-conjugated streptavidin and chemiluminescence read on a plate reader. Hits with unique amino acid sequences in the variable regions were subcloned into mammalian expression vectors with human IgG2σ or human Kappa. These antibodies were transiently transfected in HEK293F cells, and the resulting supernatants containing mAbs were characterized for their ability to bind to CD47 on Jurkat cells by FACS and to block SIRPα binding to Jurkat cells as previously described. Positive antibodies were purified from culture supernatants by Protein-A chromatography for further evaluation.

To improve affinity of C47B91, new Fab libraries were designed, with diversity introduced into the variable region of the light chain and heavy chain. The Fab libraries were constructed in a pIX phage display system as described in U.S. Pat. No. 6,472,147 and International Application No. WO09/085462. Affinity maturation Fab libraries displayed on phage coat protein IX was panned against CHO-S mammalian cells expressing different levels of full length of human CD47. The phage library was pre-cleared with CHO-S parental cells by incubating together in 10% FBS/DMEM at 4ºC overnight. About 1 x 10^7^ CHO-S CD47 expressing cells were used for 3 rounds of panning, which was about 2 hours at 4ºC for each round. Same libraries were also used to pan against biotinylated human CD47 ECD monomer. The ECD panning was performed for one hour at 25ºC for three rounds. Fab production was induced from phage plasmid DNA enriched after panning. The supernatants containing secreted Fabs were used directly to test for inhibition of recombinant human SIRPα binding to human CD47 on Jurkat cells and to check their binding to human CD47 ECD monomer and dimer by ELISA as previously described. Fab hits with unique amino acid sequences were converted to human IgG2σ/ human Kappa. These antibodies were transiently transfected in HEK293F cells, and then purified from culture supernatants by Protein-A chromatography for further characterization.

**Affinity determination:** Kinetic binding affinities to human CD47 and cyno CD47 proteins were determined by a Protein Interaction Array System (ProteOn). Briefly, mAbs were captured on the sensor chip via anti-IgG-Fc to reach surface density of 200-350 RU. The CD47 ECD monomeric proteins were serially titrated from 300 nM down to 3.7 nM and injected for 5 min. The dissociation was monitored for 30 min. Data were fitted to 1:1 Langmuir binding model.

**Epitope binning**: CD47 antibodies were individually assessed for ability to compete with the rest of the antibodies in the panel for binding to CD47 by ProteOn. The antibodies were directly immobilized on GLC sensors (BioRad, Hercules, CA). Competing antibody samples were pre-incubated in excess (10:1 antibody to CD47 ratio) with CD47 ECD protein for 4 hours before injection over the chip surface for 4 min to allow binding. Antibodies binding to CD47 at the same time theoretically recognize spatially-separated epitopes on a monomeric protein, while antibodies exhibiting reduction of activity across the entire panel are hypothesized to bind to similar epitopes.

**Preparation, crystallization, and structure determination of Fab/CD47 complexes:** Fabs of C47B161, C47B222, and B6H12.2 were expressed from transiently transfected HEK293 cells as secreted proteins with a Heavy Chain C-terminal -6xHis tag. Human CD47 ECD (mature protein residues 1-123) with a C15G mutation to prevent aggregation and a C-terminal -6x His tag was transiently expressed from HEK293S GnTI- cells as a secreted protein. The Fabs and CD47 molecules were purified via immobilized-metal affinity chromatography using a 5ml HisTrap column (GE Healthcare) followed by a final polishing size exclusion chromatography step on a 26/60 Superdex 200 column (GE Healthcare).

The C47B222 Fab / CD47 and B6H12.2 Fab / CD47 complexes were prepared by overnight mixing of CD47 and Fab at a molar ratio of 1.2:1 (excess CD47) at 4 °C, while buffer exchanging to 20 mM Tris pH 7.5. The C47B222 and B6H12.2 complexes were then eluted from a monoS 5/50 column (GE Healthcare) with a gradient of NaCl and concentrated to 17 and 3 mg/mL, respectively, for crystallization trials.

The C47B161 Fab / CD47 complex required prior deglycosylation of CD47 with 200 mU endoH/mg of CD47 (Sigma catalog number E7642) and purification of deglycosylated CD47 with a 2 mL concanavalin A Sepharose 4B column (GE Healthcare). The C47B161 complex was then prepared by overnight mixing of deglycosylated CD47 and Fab at a molar ratio of 1.2:1 (excess CD47) at 4 °C, purified by size-exclusion chromatography on a 10/300 Superdex 200 column (GE Healthcare), and concentrated to 7.5 mg/mL in 20 mM HEPES, pH 7.4, 0.1 M NaCl, 5% glycerol for crystallization trials.

Crystals of Fab/CD47 complexes were obtained using the sitting drop vapor-diffusion method with micro-seeds at 20°C. Crystallization trials were carried out in 96-well Corning 3550 plates using 320 nL drops (160 nL protein, 120 nL well and 40 nL seed solutions) that were set up with a Mosquito LCP robot (TTP Labtech) operating at room temperature. Diffraction quality crystals of each complex were obtained from the following conditions: C47B161 complex: 34% PEG 8000, 0.1M HEPES, pH 7.5; C47B222 complex: 25% PEG 3350, 1M LiCl, 0.1M MES, pH 6.5; B6H12.2 complex: 2.4 M (NH_4_)_2_SO_4_, 0.1 M sodium acetate, pH 5.5. For X-ray data collection, the crystals were soaked for a few seconds in a cryo-protectant solution containing corresponding mother liquor supplemented with 20% glycerol, and then flash frozen in liquid nitrogen. X-ray diffraction data were collected with a Dectris Pilatus 6M Pixel Array detector at beamline 17-ID of the Advanced Photon Source (APS) at Argonne National Laboratory. Diffraction data were processed with the program HKL2000.[^2^](#_ENREF_2) The structures were solved by molecular replacement with Phaser[^3^](#_ENREF_3) using the crystal structures of CD47 (PDB code 2JJS;[^4^](#_ENREF_4)) and the corresponding Fab (data not shown) as search models. The structures were refined with PHENIX[^5^](#_ENREF_5) and model adjustments were carried out using COOT.[^3^](#_ENREF_3) Epitope, paratope, and SIRPα binding residues were assigned within a 4 Å contact distance cut-off using the CCP4 program CONTACT.[^6^](#_ENREF_6) Structural overlays were performed with COOT[^3^](#_ENREF_3) and molecular graphics were generated with PyMol (PyMOL Molecular Graphics System, Version 1.4.1, Schrödinger, LLC).

The Fab/CD47 structures contain CD47 residues 1 to at least 114 of the mature protein with glycans in positions 5, 16, 32, 55, and 93, except for deglycosylated CD47 in the C47B161 complex which has fewer visible glycans. The Fab and CD47 C-terminal 6xHis tags and Fab inter-chain disulfide bond are disordered. In the case of the C47B222 Fab, heavy chain residues 134-140 are also disordered. The antibody/antigen combining site is well defined by the electron density in all three complexes, which allows reliable positioning of the binding residues. The Fabs are numbered sequentially in all Figures and CD47 numbering starts at the N-terminus of the mature protein.

The CD47 molecules in the Fab complexes superimposed among themselves and with the CD47 molecule bound to SIRPα (PDB code 2JJS;[^4^](#_ENREF_4)) with RMSD of 0.45-0.98Å (Cα atom superposition), indicating a high degree of structural similarity of CD47 in the various complexes and absence of large conformational changes induced by Fab or SIRPα binding.

**Accession numbers:**

The atomic coordinates and structure factors for C47B161/CD47 (code 5TZT), C47B222/CD47 (code 5TZ2), and B6H12.2/CD47 (code 5TZU) have been deposited in the Protein Data Bank.

**SIRPα blocking activity:** Jurkat cells expressing human CD47 were washed and resuspended in phosphate buffered saline (PBS) and then captured onto MSD 96-well high bind plates at 30,000 cells per well by incubating at 37⁰C for 1.5 hours. The plates were blocked with 15% fetal bovine serum (FBS) (or heat-inactivated FBS) for 30 min at room temperature with gentle agitation. The potency of SIRPα blocking by the CD47 antibodies was measured by serially titrating the antibodies and incubating with Jurkat cells for 1 hour, then removing unbound antibodies before addition of recombinant SIRPα-Fc to Jurkat cells and incubating for another 1.5 hrs. The bound SIRPα was detected with a MSD Sulfo-Tag labeled mouse anti-SIRPα antibody. The Electrochemiluminescence (ECL) signal was plotted as a function of antibody concentrations, and EC_50_ values were obtained from fitting the dose-response curves using non-linear regression model in GraphPad Prism.

**Hemagglutination activity:** Blood was collected from healthy donor volunteers into Vacutainer collection tubes (BD Biosciences, San Jose, CA, USA), buffered with sodium citrate. Blood was washed with PBS three times and a 2% erythrocyte suspension (v/v) was prepared in PBS. 50 µl of the serially (2-fold) diluted antibodies were incubated with 50 µl of the 2% erythrocyte suspension for 2 hours at room temperature in clear 96-well round bottom plates and subsequently were scored for hemagglutination when RBCs did not appear as tight pellets in the well.

**Platelet aggregation:** Blood was collected from healthy donor volunteers into Vacutainer collection tubes (BD Biosciences) buffered with sodium citrate. Platelet rich plasma (PRP) and platelet poor plasma (PPP) were prepared with the PDQ platelet function centrifuge (Biodata Corporation, Horsham, PA) according to the manufacturer’s protocol. Platelet aggregation was measured with the PAP-8E aggregometer (Biodata Corporation) as recommended by the manufacturer. Antibodies (or positive control, ADP) were added to PRP for a final concentration of 10 µM ADP or 200 µg/ml test antibodies. Aggregation was determined by measuring the transmission of light through the sample at 37 °C with continuous stirring. The transmission of PPP was set as 100%. Aggregation was recorded for a total of 6 minutes.

**RESULTS**

**The Epitope and Paratope Regions of C47B161, C47B222, and B6H12.2**

C47B161 recognizes a conformational epitope composed of residues in the CD47 N-terminal (residues Q1 and L3), BC (N27 and E29) and FG (L101 and T102) loops, and the F (E97) and G (R103 and E104) β-strands as seen in Supplemental Figure 3A. The antibody light chain is positioned primarily on the CD47 β-sheet, while the heavy chain covers the apical loop regions of the antigen. The C47B161 paratope is composed of residues from all CDRs except CDR-L3 (Supplemental Figure 4). In comparison to the other anti-CD47 Fabs under evaluation, C47B161 has a five-residue-longer CDR-L1. Residues Y35 and Y37 are near the tip of the CDR-L1 loop and enhance the affinity of the antibody for its antigen by hydrogen-bonding the β-sheet of CD47 (Supplemental Figure 3a). The other residues at the CDR-L1 loop tip (H31 and N33 residues) are not involved in direct contact with CD47 but, play an important role in orienting Y35 and, especially, Y37 for effective interaction with the antigen. On the heavy chain side, all CDRs are involved in interactions with the antigen, particularly with the N-terminal Q1 residue, which is seen as cyclized pyroglutamate in the Fab/CD47 structure, and the 27-29 loop segment.

C47B222 recognizes a conformational epitope composed of residues of the C (Y37 and K39), C’ (D46, T49, and D51), C’’ (K56 and T58) and F (T99) β-strands and the BC (E35), C’C’’ (A53 and L54), C’’E (V59), and FG (L101 and T102) loops as shown in Supplemental Figure 3b. In comparison to C47B161, the C47B222 epitope extends over a wider area of the CD47 β-sheet and the antibody’s heavy chain is positioned closer to the cell membrane plane (Figure 1 in the main text). Leucine 54, in the CD47 C’C’’ loop, is in a central location of the antibody-antigen combining site and contacts all three heavy chain CDRs (Supplemental Figure 3b). Similar to C47B161, the C47B222 paratope is composed of residues from all CDRs except CDR-L3 (Supplemental Figure 4) and the majority of the interactions with CD47 are made by the heavy chain. There is clear separation between the epitope regions recognized by the heavy and light chains. The heavy chain interacts exclusively with the C’C’’ loop and β-strand regions (except for the F strand), while the light chain binds the BC and FG loops and the F β-strand.

B6H12.2 recognizes a conformational epitope composed of residues of the C (V36, Y37, K39 and K41), C’ (D46 and D51), F (E97 and T99) and G (R103 and E104) β-strands and the BC (E29, Q31, N32, T34, and E35), C’C’’ (A53) and FG (E100-R102) loops as shown in Supplemental Figure 3c. The B6H12.2 epitope area is considerably larger than the areas recognized by C47B161 and C47B222. The epitope regions of all three antibodies overlap with each other. The B6H12.2 paratope is composed of residues from all CDRs (Supplemental Figure 4) and the antibody / antigen recognition appears to be mostly driven by hydrogen-bonds.

**FIGURE LEGENDS**

**Figures 1: Hemagglutination by anti-CD47 antibodies. (**a) Hemagglutination of human red blood cells in response to varying doses of anti-human CD47 monoclonal antibodies B6H12.2 (cloned into IgG1 and IgGσ) and commercially available BRIC126. (b) Representative results of hemagglutination assays with 23 unique anti-CD47 mAbs. Shown are hemagglutination results in response to C47B91, C47B98, C47B116, C47B123, and C47B131. (c) Hemagglutination of human red blood cells in response to varying doses of anti-human IgG1/IgG2σ C47B157, C47B161, and C47B222. IgG2σ C47B98 known to induce hemagglutination (see 1b) was included as positive control.

**Figures 2:** **Platelet aggregation by anti-CD47 antibodies.** Aggregation of human platelets in response to incubation of platelet rich plasma with (a) PBS, 200 µg/ml IgG1/IgG2σ B6H12.2 and 10 µM ADP; (b) PBS, 200 µg/ml IgG1/IgG2σ C47B157, and IgG1 B6H12.2; (c) PBS, 200 µg/ml IgG1/IgG2σ C47B161, and IgG1 B6H12.2; and (d) PBS, 200 µg/ml IgG1/IgG2σ C47B222, and IgG1 B6H12.2.

**Figure 3: Epitope location and antibody interactions with human CD47.** The epitope regions of C47B161 (panel a), C47B222 (panel b), and B6H12.2 (panel c) are shown in purple on the pink cartoon representation of CD47 from each Fab / CD47 structure. Fab light and heavy chains are shown in cyan and green, respectively, and hydrogen bonds as yellow dashed lines.

**Figure 4:** **Paratope residues of C47B161, C47B222, and B6H12.2.** Only the sequences for the variable domain light (VL) and heavy (VH) chains are shown. The paratope residues are shaded and the CDR regions are underlined (Kabat definition).

**Figure 5: IgG1 anti-CD47 mAbs induce phagocytosis of Jurkat cells. (**a) CD47 expression on Jurkat cells. **(**b) Phagocytosis of Jurkat cells by human PBMC derived macrophages in response to the indicated concentrations of IgG1/IgG2σ anti-CD47 mAbs was assessed by flow cytometry. (c) CD47 expression on HL60, Kasumi-3, and MV4-11 cells.

**REFERENCES**

1. Shi L, Wheeler JC, Sweet RW, Lu J, Luo J, Tornetta M*, et al.* De novo selection of high-affinity antibodies from synthetic fab libraries displayed on phage as pIX fusion proteins. *Journal of molecular biology* 2010 Mar 26; **397**(2)**:** 385-396.

2. Otwinowski Z, Minor W. Processing of X-ray diffraction data collected in oscillation mode. *Methods in Enzymology* 1997; **276:** 307-326.

3. Emsley P, Cowtan K. Coot: model-building tools for molecular graphics. *Acta crystallographica Section D, Biological crystallography* 2004 Dec; **60**(Pt 12 Pt 1)**:** 2126-2132.

4. Hatherley D, Graham SC, Turner J, Harlos K, Stuart DI, Barclay AN. Paired receptor specificity explained by structures of signal regulatory proteins alone and complexed with CD47. *Molecular cell* 2008 Jul 25; **31**(2)**:** 266-277.

5. Adams PD, Gopal K, Grosse-Kunstleve RW, Hung LW, Ioerger TR, McCoy AJ*, et al.* Recent developments in the PHENIX software for automated crystallographic structure determination. *Journal of synchrotron radiation* 2004 Jan 1; **11**(Pt 1)**:** 53-55.

6. Collaborative Computational Project N. The CCP4 suite: programs for protein crystallography. *Acta crystallographica Section D, Biological crystallography* 1994 Sep 1; **50**(Pt 5)**:** 760-763.
