# Supplementary material for: Anti-leukemic activity and tolerability of anti-human CD47 monoclonal antibodies
Source: Blood Cancer J. 2017 Feb 24;7(2):e536–. doi: 10.1038/bcj.2017.7 (PMC5386341; doi:10.1038/bcj.2017.7)
Supplement: Supplementary Table 1 [file bcj20177x2.docx]

**Supplementary Table 1: AML patient sample characteristics**

| Patient | AML  Diagnosis | Sex | Age | Race | Cytogenetics | WBC (10E9/L) | % Blasts (BM) | Flt3 |  |
| --- | --- | --- | --- | --- | --- | --- | --- | --- | --- |
| AML-1 | Secondary/  relapsed | M | 64 | Caucasian | 47,XY,+8[2]/46,XY,t(3;17)(q21;q25)[2]/46,XY[16] | 71.1 | 87 | ITD |  |
| AML-2 | biphenotypic | M | 78 | NA | 46,XY,der(7)?t(7;13)(q22;q13)[13]/48,idem,+9,+13[7]/47,XY,+12[1]/48,XY,+12,+13[1] | 245.7 | NA | ITD |  |
