# Supplementary material for: Anti-leukemic activity and tolerability of anti-human CD47 monoclonal antibodies
Source: Blood Cancer J. 2017 Feb 24;7(2):e536–. doi: 10.1038/bcj.2017.7 (PMC5386341; doi:10.1038/bcj.2017.7)
Supplement: Supplementary Table 2 [file bcj20177x3.docx]

**Supplementary Table 2: AML cell line characteristics**

| Cell line | Established at | Sex | Age | Race | Cytogenetics | FAB | FLT3 |  |
| --- | --- | --- | --- | --- | --- | --- | --- | --- |
| HL60 | Diagnosis | F | 35 | Caucasian | hypotetraploid karyotype without clear mode, 1.5% polyploidy; 82(78-88)<4n>XX, -X, -X, -2, -3, -4, -5, -8, -9, -10, -14, -16, -17, -17, +3m, der(6)t(6;?)(q25;?)/dup(6)(q23;qter)x2, del(9)(p22), del(11)(q22/23), der(16)t(16;17)) | M2 | WT |  |
| Kasumi-3 | Diagnosis | M | 57 | Asian | t(3;7)(q27:q22), del(5)(q15), del(9)(q32), add(12)(p11) | M0 | WT |  |
| MV4-11 | Diagnosis | M | 10 | Caucasian | hyperdiploid karyotype - 48(46-48)<2n>XY, +8, +18, +19, -21, t(4;11)(q21;q23) | M5 | ITD |  |
