# Supplementary material for: Anti-leukemic activity and tolerability of anti-human CD47 monoclonal antibodies
Source: Blood Cancer J. 2017 Feb 24;7(2):e536–. doi: 10.1038/bcj.2017.7 (PMC5386341; doi:10.1038/bcj.2017.7)
Supplement: Supplementary Table 3 [file bcj20177x4.docx]

**Supplementary Table 3: Anti-CD47 mAb characteristics**

| Ab ID | Epitope Bin | Human CD47 K_D_ (nM) | Cyno CD47 K_D_ (nM) | K_D_ Ratio Cyno/ Human | SIRPα-blocking EC_50_ (μg/ml) | Hemagglutination |
| --- | --- | --- | --- | --- | --- | --- |
| B6H12.2* | 1 | 0.5 | 0.6 | 1.1 | 0.21 | N |
| C47B120 | 1 | 5.6 | 78.5 | 14.1 | 0.26 | N |
| C47B91 | 1 | 15.6 | 24.5 | 1.6 | 0.06 | N |
| C47B116 | 2 | 1.4 | 1.0 | 0.7 | 0.1 | N |
| C47B119 | 2 | 2.5 | 2.4 | 1 | 0.32 | N |
| C47B125 | 2 | 40.5 | 33.2 | 0.8 | not fitted | N |
| C47B131 | 3 | 1.5 | 162.0 | 111.7 | 0.33 | N |
| C47B121 | 3 | 7.2 | no binding | - | 0.2 | N |
| C47B117 | 4 | 87.2 | 152.0 | 1.7 | 2.96 | N |
| C47B126 | 1 | 0.3 | 0.3 | 0.8 | 0.04 | Y |
| C47B128 | 1 | 2.2 | 2.2 | 1 | 0.05 | Y |
| C47B130 | 1 | 4.8 | 4.3 | 0.9 | 0.11 | Y |
| C47B96 | 1 | 60.8 | 35.8 | 0.6 | 0.46 | Y |
| C47B133 | 2 | 1.1 | 3.8 | 3.6 | 0.13 | Y |
| C47B1 | 2 | 1.4 | 7.1 | 5 | 0.36** | Y |
| C47B122 | 2 | 1.6 | 2.9 | 1.8 | 0.08 | Y |
| C47B123 | 2 | 2.1 | 1.7 | 0.8 | 0.07 | Y |
| C47B134 | 2 | 2.5 | 2.7 | 1.1 | 0.47** | Y |
| C47B132 | 2 | 2.7 | 9.6 | 3.6 | 0.58** | Y |
| C47B129 | 2 | 2.8 | 4.9 | 1.8 | 0.12 | Y |
| C47B98 | 2 | 3.2 | 3.2 | 1 | 0.95 | Y |
| C47B127 | 2 | 3.3 | 4.1 | 1.3 | 0.19 | Y |
| C47B124 | 2 | 3.7 | 2.9 | 0.8 | 0.11 | Y |
| C47B118 | 2 | 4.0 | 2.6 | 0.7 | 0.09 | Y |
