# Supplementary material for: Anti-leukemic activity and tolerability of anti-human CD47 monoclonal antibodies
Source: Blood Cancer J. 2017 Feb 24;7(2):e536–. doi: 10.1038/bcj.2017.7 (PMC5386341; doi:10.1038/bcj.2017.7)
Supplement: Supplementary Table 4 [file bcj20177x5.docx]

**Supplementary Table 4: Data collection and refinement statistics**

| **Complex** | **C47B161** | **C47B222** | **B6H12.2** |
| --- | --- | --- | --- |
| **Data collection** |  |  |  |
| Resolution limit (Å)^a^ | 2.90 (2.95-2.90) | 2.30 (2.34-2.30) | 2.10 (2.14-2.10) |
| Space group | P2_1_ | P2_1_ | C2 |
| Unit cell dimensions |  |  |  |
| *a, b, c* (Å) | 74.6, 60.9, 124.1 | 60.6, 72.9, 72.7 | 161.8, 54.5, 83.5 |
| α, β, γ (°) | 90, 90, 90 | 90, 109, 90 | 90, 96, 90 |
| Asymmetric unit | 2 complexes | 1 complex | 1 complex |
| No. measured reflections | 77,044 | 82,617 | 130,229 |
| Completeness (%)^a^ | 98.6 (90.9) | 98.8 (97.0) | 98.7 (99.1) |
| Redundancy^a^ | 3.1 (2.9) | 3.1 (2.8) | 3.1 (3.0) |
| R_sym_ (%)^a, b^ | 11.9 (46.5) | 8.3 (43.2) | 10.5 (36.8) |
| I/σ(I)^a^ | 9.1 (2.0) | 12.8 (2.4) | 10.8 (4.2) |
| **Refinement** |  |  |  |
| Resolution limit (Å) | 2.9 | 2.3 | 2.1 |
| No. reflections in working set | 23,559 | 24,888 | 39,864 |
| No. reflections in test set | 1,273 | 1,329 | 2,129 |
| No. atoms (protein/water/other)^c^ | 8,010 / 118 / 30 | 4,086 / 105 / 6 | 4,192 / 333 / 28 |
| R_factor_ / R_free_ (%)^d^ | 25.7 / 32.8 | 20.1 / 25.5 | 17.7 / 21.3 |
| RMSD bond lengths (Å)^e^ | 0.005 | 0.002 | 0.005 |
| RMSD bond angles (°)^e^ | 1.016 | 0.556 | 0.863 |
| B-factors (Å^2^; protein/water/other)^c^ | 70.2 / 42.7 / 93.0 | 67.1 / 66.2 / 88.6 | 36.6 / 42.6 / 74.2 |
| Ramachandran favored (%) | 95.1 | 95.3 | 98.3 |
| Ramachandran outliers (%) | 0.2 | 0.0 | 0.0 |

^a^ Numbers in parentheses refer to the highest resolution shell.

^b^ R_sym_ = 100 x (Σ_hkl_Σ_i_|I(*hkl;i*) – <I(hkl)>| / (Σ_hkl_Σ_i_I(hkl;i)], where I(*hkl;i*) is the intensity of an individual measurement of a reflection and <I(*hkl*)> is the average intensity of that reflection.

^C^ Other refers to glycerol in the C47B161 and C47B222 complexes and glycerol, sulfate, and acetate in the B6H12.2 complex.

^d^ R_factor_ = 100 x (Σ_hkl_||F_obs_| − |F_calc_|| / (Σ_hkl_|F_obs_|, where |F_obs_| and |F_calc_| are the observed and calculated structure factor amplitudes. R_free_ equals the R_factor_ of test set (5% of the data removed prior to refinement).

^e^ RMSD is the root mean square deviation.
