# Supplementary material for: Anti-leukemic activity and tolerability of anti-human CD47 monoclonal antibodies
Source: Blood Cancer J. 2017 Feb 24;7(2):e536–. doi: 10.1038/bcj.2017.7 (PMC5386341; doi:10.1038/bcj.2017.7)
Supplement: Supplementary Table 5 [file bcj20177x6.docx]

**Supplementary Table 5: Mean number of red blood cells in cynomolgus monkeys**

| **Treatment** | **Red Blood Cells** | Study Day | | | | | | | | | |
| --- | --- | --- | --- | --- | --- | --- | --- | --- | --- | --- | --- |
|  |  | Prestudy | 3 | 5 | 8* | 10 | 12 | 15 | 21 | 26 | 31 |
| **PBS Control** | Number x 10^6^/µL | 6.21 | 5.56 | 5.44 | 5.23 | 4.88 | 5.03 | 4.88 | 5.28 | 5.37 | 5.46 |
|  |  |  |  |  |  |  |  |  |  |  |  |
| **IgG2σ C47B222-(CHO)** | Number x 10^6^/µL | 5.69 | 4.90 | 4.69 | 4.57 | 4.32 | 4.25 | 4.36 | 4.86 | 5.09 | 5.30 |
|  | (% control) | (88.1%) | (86.3%) | (87.4%) | (88.4%) | (84.6%) | (89.4%) | (91.9%) | (94.9%) | (97.1%) | (97.1%) |
|  |  |  |  |  |  |  |  |  |  |  |  |
| **IgG1 C47B222-(CHO)** | Number x 10^6^/µL | 5.83 | 4.42 | 3.30 | 3.05 | 2.23 | 2.19 | 2.76 | 3.85 | 4.24 | 4.59 |
|  | (% control) | (93.8%) | (79.4%) | (60.6%) | (58.4%) | (45.7%) | (43.6%) | (56.6%) | (72.8%) | (79.1%) | (84.1%) |
| Female cynomolgus monkeys (n = 4/group) were treated by IV injection on study days 1 and 8.  IgG2σ C47B222-(CHO)-treated animals received 1 mg/kg on study day 1 and 10 mg/kg on study day 8.  IgG1 C47B222-(CHO)-treated animals received 1 mg/kg on study days 1 and 8.  * = Blood was collected for hematology prior to treatment on study day 8. | | | | | | | | | | | |
