# Supplementary material for: Anti-leukemic activity and tolerability of anti-human CD47 monoclonal antibodies
Source: Blood Cancer J. 2017 Feb 24;7(2):e536–. doi: 10.1038/bcj.2017.7 (PMC5386341; doi:10.1038/bcj.2017.7)
Supplement: Supplementary Figure 1 [file bcj20177x7.ppt]

## Slide 1
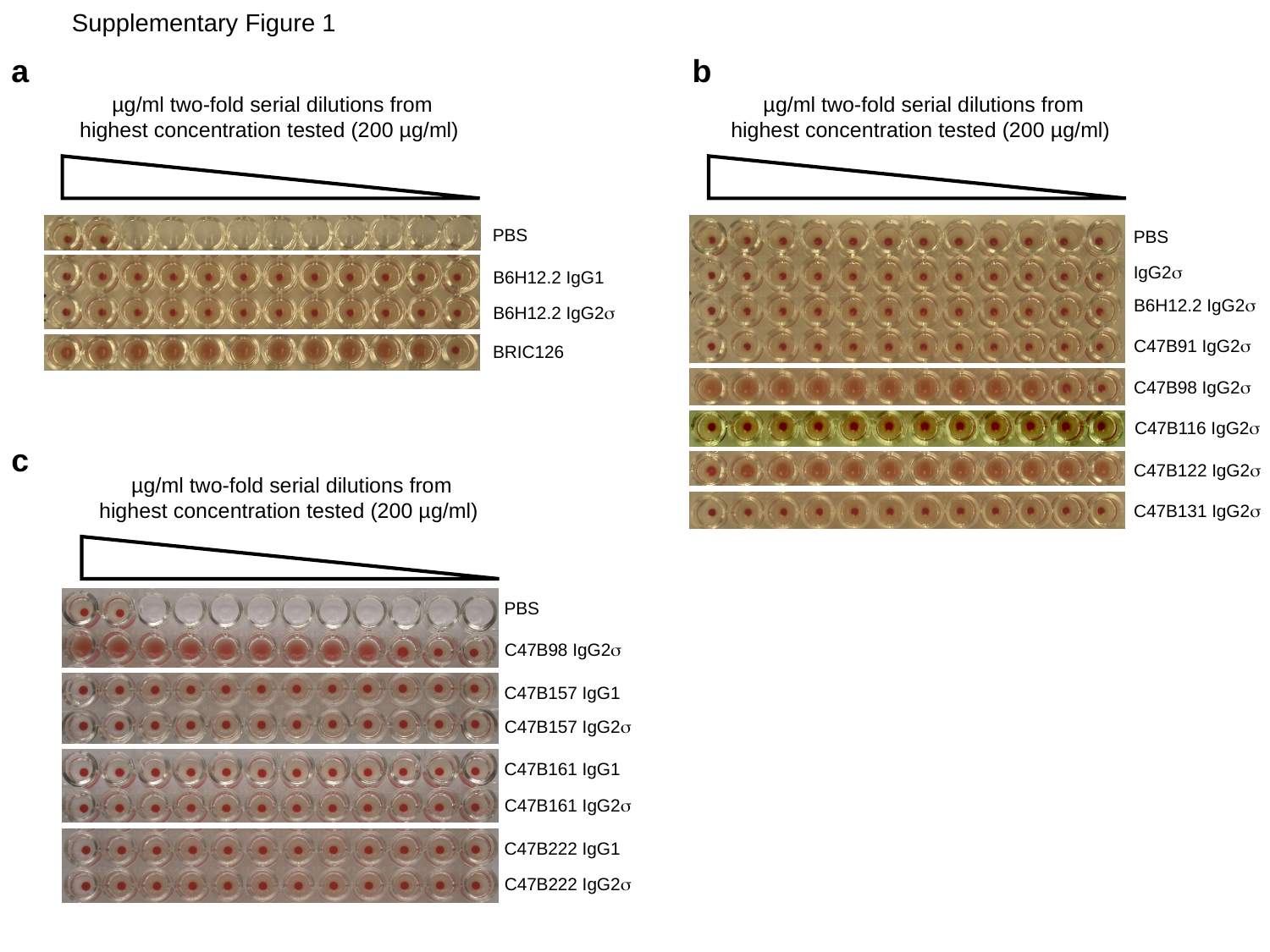

Supplementary Figure 1
a
b
µg/ml two-fold serial dilutions from highest concentration tested (200 µg/ml)
µg/ml two-fold serial dilutions from highest concentration tested (200 µg/ml)
PBS
PBS
IgG2
B6H12.2 IgG1
B6H12.2 IgG2
B6H12.2 IgG2
C47B91 IgG2
BRIC126
C47B98 IgG2
C47B116 IgG2
c
C47B122 IgG2
µg/ml two-fold serial dilutions from highest concentration tested (200 µg/ml)
C47B131 IgG2
PBS
C47B98 IgG2
C47B157 IgG1
C47B157 IgG2
C47B161 IgG1
C47B161 IgG2
C47B222 IgG1
C47B222 IgG2
