# Supplementary material for: Anti-leukemic activity and tolerability of anti-human CD47 monoclonal antibodies
Source: Blood Cancer J. 2017 Feb 24;7(2):e536–. doi: 10.1038/bcj.2017.7 (PMC5386341; doi:10.1038/bcj.2017.7)
Supplement: Supplementary Figure 2 [file bcj20177x8.ppt]

## Slide 1
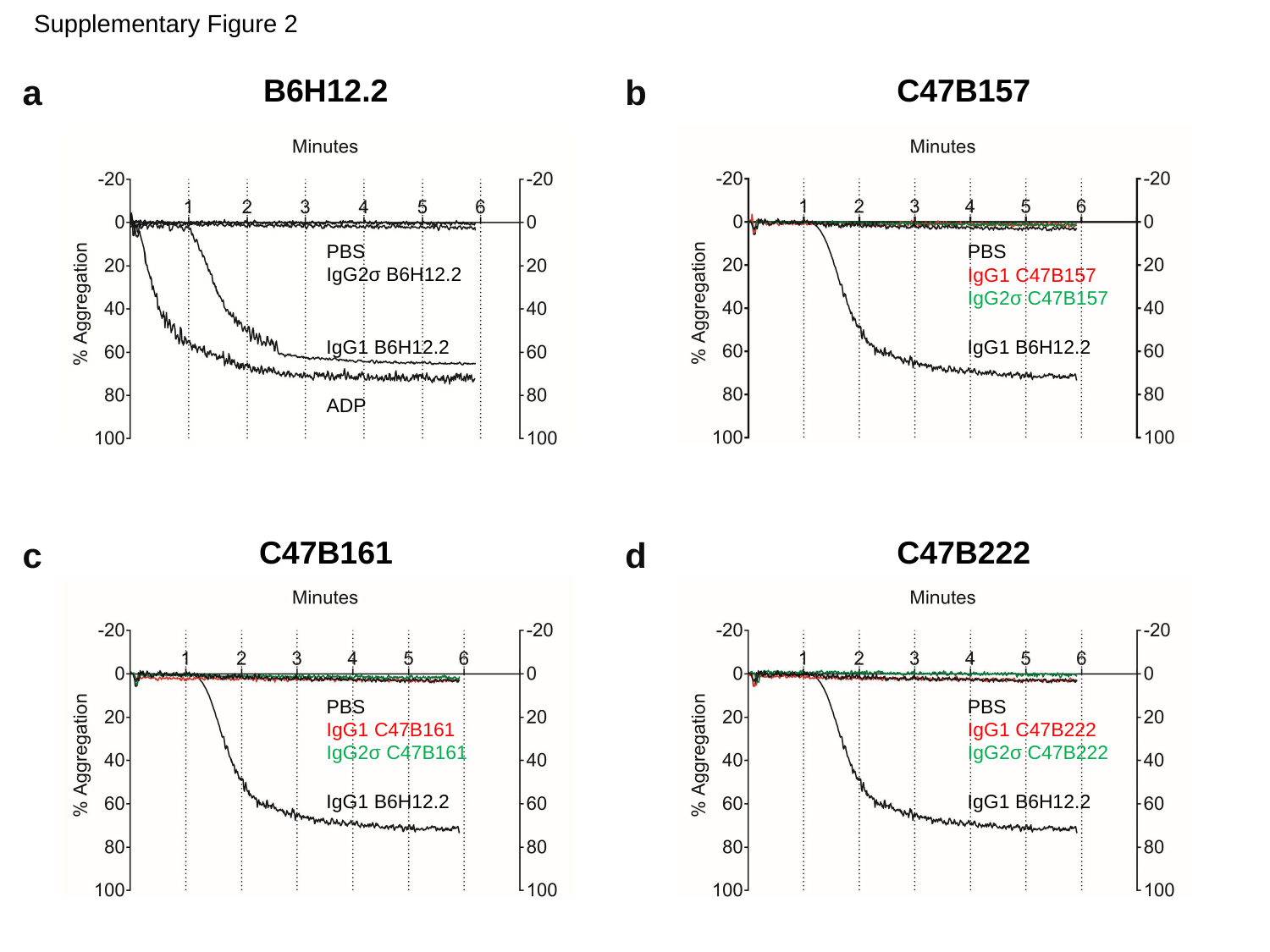

Supplementary Figure 2
a
a
B6H12.2
b
b
C47B157
PBS
IgG2σ B6H12.2
PBS
IgG1 C47B157
IgG2σ C47B157
IgG1 B6H12.2
IgG1 B6H12.2
ADP
c
c
C47B161
d
d
C47B222
PBS
IgG1 C47B161
IgG2σ C47B161
PBS
IgG1 C47B222
IgG2σ C47B222
IgG1 B6H12.2
IgG1 B6H12.2
