# Supplementary figures and images for: Anti-leukemic activity and tolerability of anti-human CD47 monoclonal antibodies
Source: Blood Cancer J. 2017 Feb 24;7(2):e536–. doi: 10.1038/bcj.2017.7 (PMC5386341; doi:10.1038/bcj.2017.7)

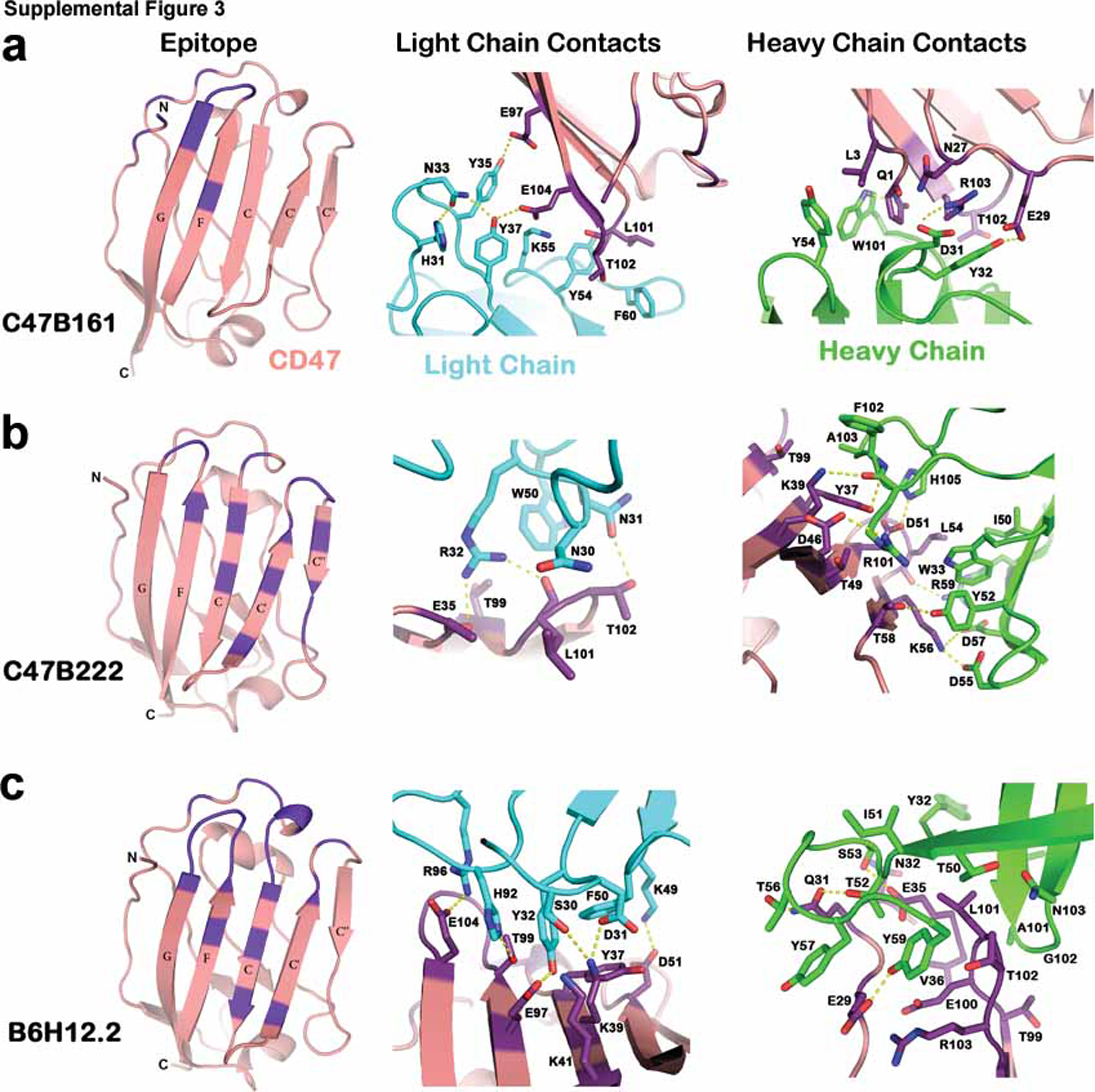

Supplement: Supplementary Figure 3 [file bcj20177x9.tif]

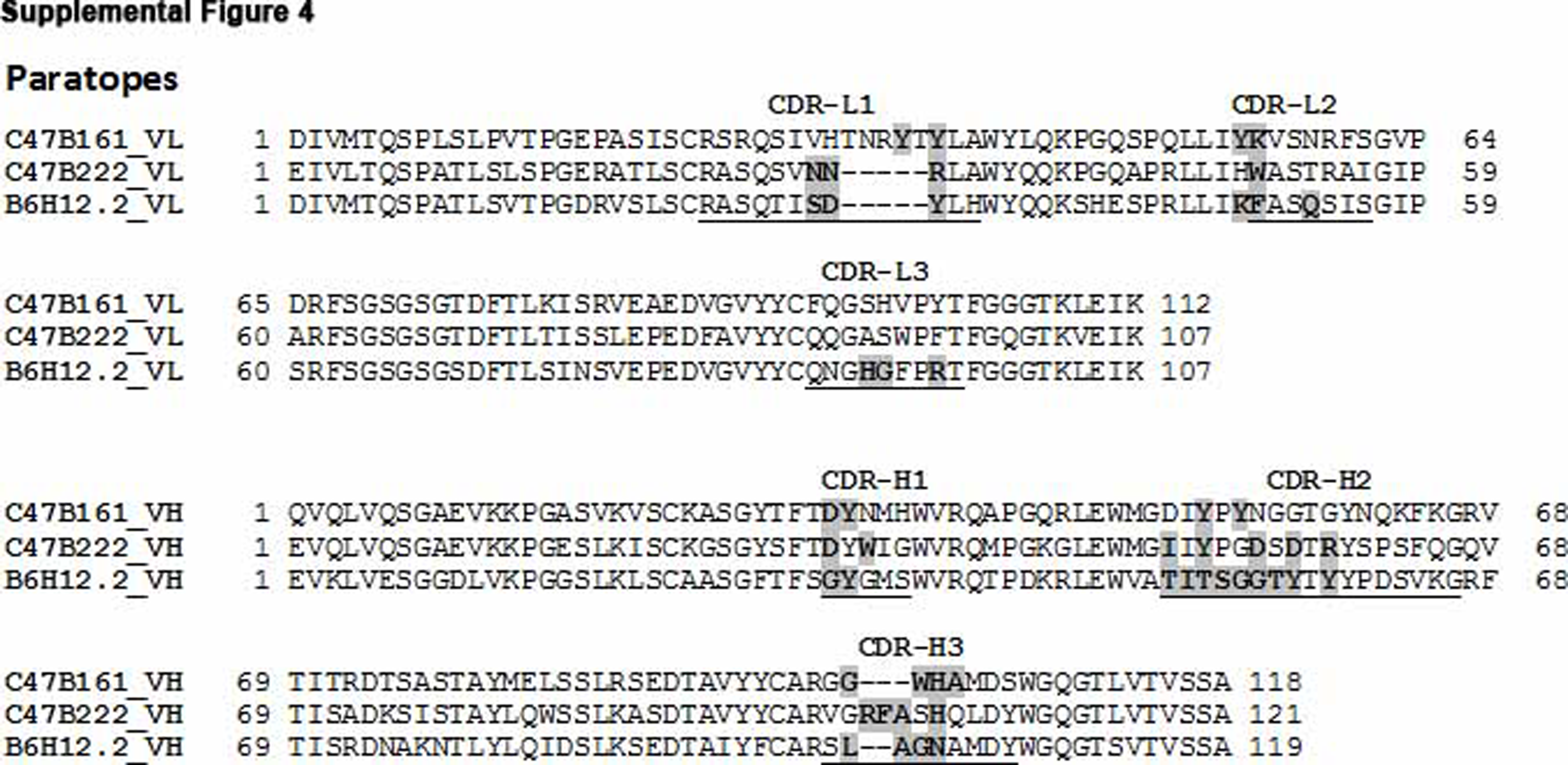

Supplement: Supplementary Figure 4 [file bcj20177x10.tif]
