# Supplementary material for: Anti-leukemic activity and tolerability of anti-human CD47 monoclonal antibodies
Source: Blood Cancer J. 2017 Feb 24;7(2):e536–. doi: 10.1038/bcj.2017.7 (PMC5386341; doi:10.1038/bcj.2017.7)
Supplement: Supplementary Figure 5 [file bcj20177x11.ppt]

## Slide 1
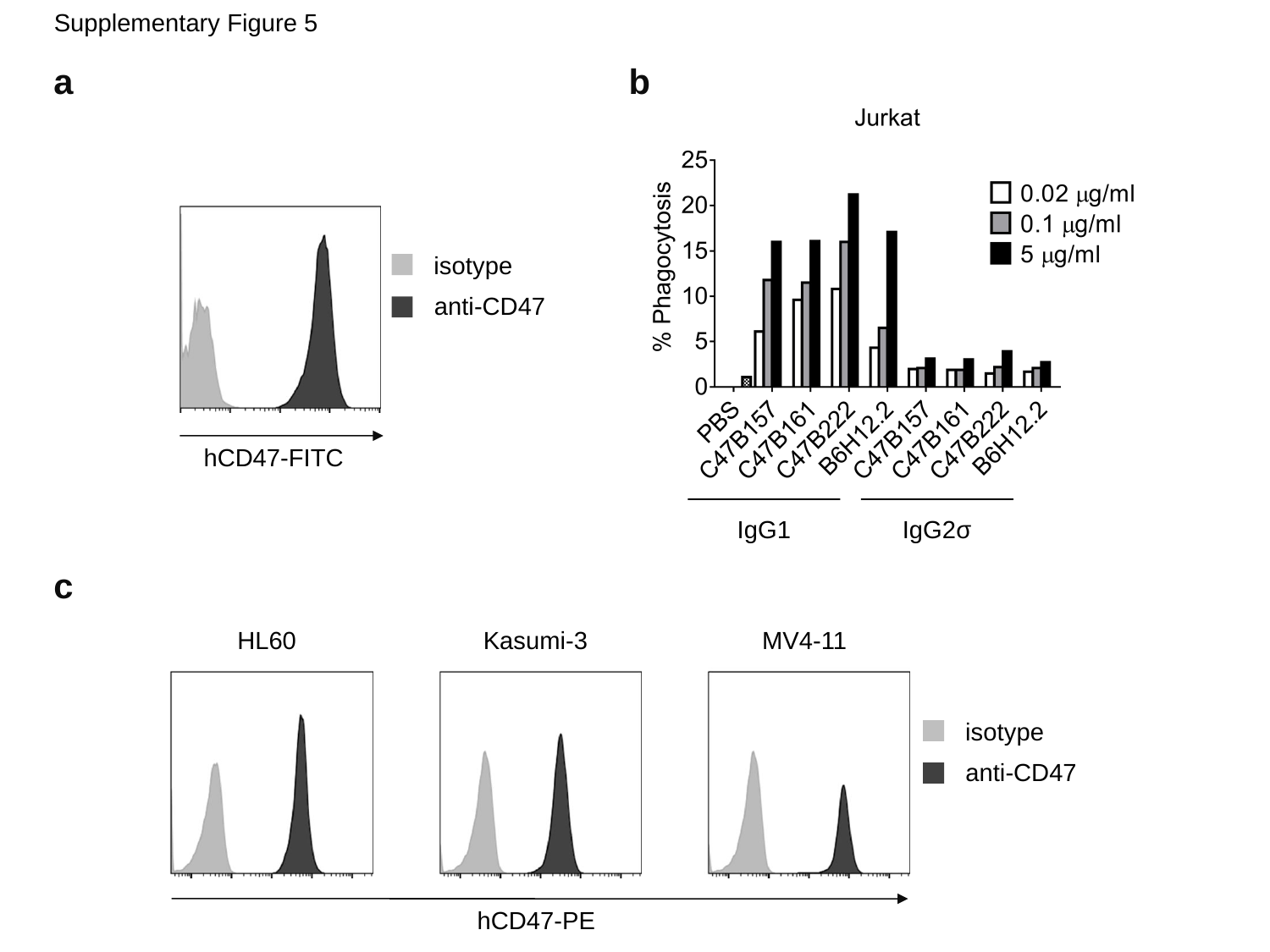

Supplementary Figure 5
a
b
isotype
anti-CD47
hCD47-FITC
IgG1
IgG2σ
c
HL60
Kasumi-3
MV4-11
isotype
anti-CD47
hCD47-PE
